# The m^6^A RNA Modification Quantity and mRNA Expression Level of RNA Methylation-Related Genes in Head and Neck Squamous Cell Carcinoma Cell Lines and Patients

**DOI:** 10.3390/biom11060908

**Published:** 2021-06-18

**Authors:** Kamila Romanowska, Agnieszka A. Rawłuszko-Wieczorek, Łukasz Marczak, Agnieszka Kosińska, Wiktoria M. Suchorska, Wojciech Golusiński

**Affiliations:** 1 The Greater Poland Cancer Centre, Department of Head and Neck Surgery, Poznan University of Medical Sciences, 61-866 Poznan, Poland; agnieszka.kosinska@wco.pl (A.K.); wgolus@ump.edu.pl (W.G.); 2 Department of Histology and Embryology, Poznan University of Medical Sciences, 60-781 Poznan, Poland; arawluszko@ump.edu.pl; 3 Radiobiology Laboratory, The Greater Poland Cancer Centre, Department of Medical Physics, Poznan University of Medical Sciences, 61-866 Poznan, Poland; wiktoria.suchorska@wco.pl; 4 European Center for Bioinformatics and Genomics Institute of Bioorganic Chemistry, Polish Academy of Sciences, 61-704 Poznan, Poland; lukasmar@ibch.poznan.pl

**Keywords:** head and neck squamous cell carcinoma, RNA methylation, m^6^A RNA modification

## Abstract

RNA methylation at the nitrogen sixth of adenosine (m^6^A, *N*^6^-methyladenosine) is the most abundant RNA modification which plays a crucial role in all RNA metabolic aspects. Recently, m^6^A modification has been assigned to mediate the biological processes of cancer cells, but their significance in HNSCC development is still poorly described. Thus, the main aim of this study was to globally quantify m^6^A modification by the mass spectrometry approach and determine the mRNA expression level of selected m^6^A RNA methyltransferase (METTL3), demethylase (FTO), and m^6^A readers (YTHDF2, YTHDC2) in 45 HNSCC patients and 4 cell lines (FaDu, Detroit 562, A-253 and SCC-15) using qPCR. In the results, we have not observed differences in the global amount of m^6^A modification and the mRNA level of the selected genes between the cancerous and paired-matched histopathologically unchanged tissues from 45 HNSCC patients. However, we have found a positive correlation between selected RNA methylation machinery genes expression and m^6^A abundance on total RNA and characterized the transcript level of those genes in the HNSCC cell lines. Moreover, the lack of global m^6^A differences between cancerous and histopathologically unchanged tissues suggests that m^6^A alterations in specific RNA sites may specifically influence HNSCC tumorigenesis.

## 1. Introduction

HNSCC (head and neck squamous cell carcinoma) arises from the epithelial membranes of the oral cavity, hypopharynx, oropharynx, nasopharynx, or larynx [1]. Currently, HNSCC is the eighth most common malignant cancer worldwide and its comprehensive treatment includes surgery, chemotherapy, and/or radiotherapy based on the tumor localization and clinical stage. Although the therapy has been refined in recent decades, the 5-year overall survival rate remains unchanged at a level of approximately 50% [2]. Furthermore, the treatment response and clinical outcome of HNSCC patients can be different even in the same tumor localization and staging. This observation is an effect of the diverse genetic and epigenetic landscape of individual tumors, which leads to an imbalance of cellular pathways and disorders in the signaling cascade and immune system [3,4,5]. Thus, the description of molecular mechanisms and identification of prognostic biomarkers for HNSCC is crucial to develop novel targeted therapeutic strategies. 

RNA methylation at the nitrogen sixth of adenosine (m^6^A, *N*^6^-methyladenosine) is the most abundant, internal RNA modification, found within the 5′-RRACH-3′ (R, purine; A* methylable A; C, cytosine; H, non-guanine base) motif, identified on almost every type of RNA [6]. Although the m^6^A is commonly enriched in the coding sequences and near translation stop codons in 3′UTR, its distribution may vary depending on the individual RNA and tissue type [7]. M^6^A modification is regulated by the dynamic interaction of RNA methylation writers (methyltransferases), erasers (demethylases), and readers (binding proteins) [8]. The METTL3 (Methyltransferase like 3) is the main catalytic core of the methyltransferase complex and works together with METTL14 (Methyltransferase like 14), WTAP (WT1 associated protein), RBM15 (RNA-binding motif protein 15), and VIRMA (Vir like m^6^A methyltransferase associated) to install m^6^A onto RNA. The demethylation is processed by FTO (fat mass and obesity-associated protein) and ALKBH5 (AlkB homolog 5), whereas direct m^6^A readers belong to the YTH domain-containing family, including YTHDF1, YTHDF2, and YTHDF3 (*N*^6^-metyladenosine RNA binding protein 1,2,3) and YTHDC1 and YTHDC2 (YTH domain containing 1, 2), which binds m^6^A depending on its location on the RNA. The m^6^A modification is involved in nearly all crucial RNA metabolic aspects, including pre-mRNA processing, mRNA splicing, translation, nuclear export, decay, and the promotion of miRNA biogenesis [9,10]. Hence, multiple studies indicate its regulatory role in many biological processes in both physiological and pathological conditions, such as cancers. RNA methylation disturbances may promote the proliferation, invasion, and migration of tumor cells and modulate the treatment response [11,12,13].

To date, only a few studies indicated the essential regulatory role of m^6^A post-transcriptional modification and RNA methylation-related genes in head and neck cancers [14,15,16,17,18]. In brief, our study quantifies the global m^6^A level in HNSCC cell lines and cancerous and paired-matched histopathologically unchanged tissues and correlates its content with expression of selected RNA methylation methyltransferase (METTL3), demethylase (FTO), and m^6^A binding proteins (YTHDF2, YTHDC2). 

## 2. Materials and Methods

### 2.1. Patient Material

Head and neck squamous cell carcinoma tissues and paired-matched histopathologically unchanged tissues were collected from 45 patients who underwent tumor surgical resection in the Department of Head and Neck Surgery, Poznan University of Medical Sciences, The Greater Poland Cancer Centre. Samples were immediately frozen and stored at −80 °C until RNA isolation. The criteria that excluded patients from this study involved second distant metastasis and an HPV-positive test result. The characterization of the total study cohort is presented in Table 1. The procedures were approved by the Local Ethical Committee of Poznań University of Medical Sciences. 

### 2.2. Cell Culture

The FaDu, Detroit 562, A-253, and SCC-15 cell lines were obtained from the Head and Neck Cancer Panel of the American Type Culture Collection (ATCC^®^TCP-1012™) and correspond with different tumor locations: hypopharynx, pharynx, salivary gland, and tongue, respectively. The FaDu cells were cultured in Dulbecco’s modified Eagle’s Medium (DMEM) (Biowest, Nuaillé, France), the Detroit 562 in Eagle’s Minimum Essential Medium (EMEM) (Biowest, Nuaillé, France), and the A-253 cells in McCoy’s 5a Medium Modified (Biowest, Nuaillé, France). The SCC-15 cells were cultured in a 1:1 mixture of DMEM and Ham’s F12 Medium (Biowest, Nuaillé, France) containing 1.2 g/L sodium bicarbonata, 2.5 Mm L-glutamine, 15 mM HEPES, and 0.5 mM sodium pyruvate supplemented with 400 ng/mL hydrocortisone. All growth media were supplemented with 10% fetal bovine serum (FBS) (Biowest, Nuaillé, France) and 1% penicillin/streptomycin (Biochrom, Holliston, MA, USA). The cell lines were cultured in an incubator at 37 °C, 5% CO_2_ atmosphere, and a humidity level of 100%. These cells were used for total RNA isolation.

### 2.3. RNA Digestion and LC-ESI-MS/MS Analysis

In total, 500 ng of RNA samples was denatured for 15 min at 95 °C and fully digested by S1 nuclease (180U, Promega, Madison, WI, USA) for 4 h at 37 °C. After the addition of alkaline phosphatase (CIAP, 30U, Promega, Madison, WI, USA) and Venom phosphodiesterase I type IV (0.01U, Sigma-Aldrich, Saint Louis, MO, USA), the samples were incubated for another 1 h at 37 °C. The digested RNA samples were diluted in 100 µL Mili-Q water (final volume 200 µL) and extracted twice by equal volume of chloroform. Afterward, the resulting aqueous layers were collected, lyophilized to dryness, and redissolved in 100 µL Mili-Q water. LC-ESI-MS/MS (liquid chromatography electrospray ionization tandem mass spectrometric) analysis of nucleosides was performed on a Shimadzu LCMS8060 mass spectrometer (Shimadzu, Japan) connected to a Nexera Mikros HPLC system (Shimadzu, Japan). Separation was carried out on a Waters NanoACQUITY Symmetry C18 column, 150 × 0.3 mm, 3.5 µm particle size. As mobile phases, formic acid in water (0.1%, *v*/*v*) and formic acid in methanol (0.1%, *v*/*v*) were used (phases A and B, respectively). Elution started with 5% solvent B for 5 min, then was raised to 30% solvent B in 10 min and to 50% solvent B in 5 min; after 3 min, the equilibration solvent ratio was set back to 5% solvent B for another 10 min. The flow rate was 5 µL/min, and the column temperature was 30 °C. The MS detection was performed under positive electrospray ionization mode, and the source parameters were as follows: probe voltage 2.6 kV, desolvation line temperature 250 °C, heater block 400 °C, nebulizing gas 1L/min. The triplequad analyzer was operated in MRM mode, and the specific reaction for m^6^A fragmentation was monitored (282.1→150.1), as well as for A (268.1→136.1), which was used for reference. All mass chromatograms were analyzed employing Shimadzu Lab Solutions software. Quantitation was based on calibration curves, which were constructed by plotting the mean peak area ratio of m^6^A/A versus the mean molar ratio of m^6^A/A (ranging from 0.05 to 5% of m^6^A/A) (Figure S1).

### 2.4. RNA Isolation, Reverse Transcription, and Real-Time Quantitative Polymerase Chain Reaction (RQ-PCR) Analysis 

The RNA purification kit (RNeasy Mini Kit, Qiagen, Hilden, Germany) was used to extract total RNA from tissue specimens. The cDNA was synthesized with RevertAid First Strand cDNA Synthesis Kit (ThermoFisher, Waltham, MA, USA) using 500 ng of total RNA, oligo dT primers, and random hexamer primers. The real-time quantitative polymerase chain reaction for individual genes expression analysis was conducted with a PowerTrack SYBR Green Master Mix (ThermoFisher, Waltham, MA, USA) using the CFX96 Real-Time System (Bio-Rad, Hercules, CA, USA). The reaction conditions for all amplicons were as follows: initially 95 °C for 15 min, followed by 40 cycles at 95 °C for 10 s, 60–62 °C (depending on the primers used) for 10 s, and 72 °C for 10 s. The gene expression was normalized to the GAPDH housekeeping gene, and relative expression levels were determined by the Pfaffl method. The primers sequence used in this study are listed in Table S1.

### 2.5. Statistical Analysis

The normality of the observed patient data distribution was assessed using the Shapiro–Wilk test. The median values were compered using the U Mann–Whitney test, and the mean values were compered using an unpaired *t*-test with Welch’s correction. The correlation between the studied variables was determined using the Spearman correlation. The patient’s survival analyses were estimated by the Kaplan–Meier method. Statistical analysis was performed with the GraphPad Prism 9.0.1 software, and *p* < 0.05 was considered statistically significant.

## 3. Results

### 3.1. Investigation of m^6^A Modification Level in HNSCC Patients and Cell Lines

**Figure 1:**
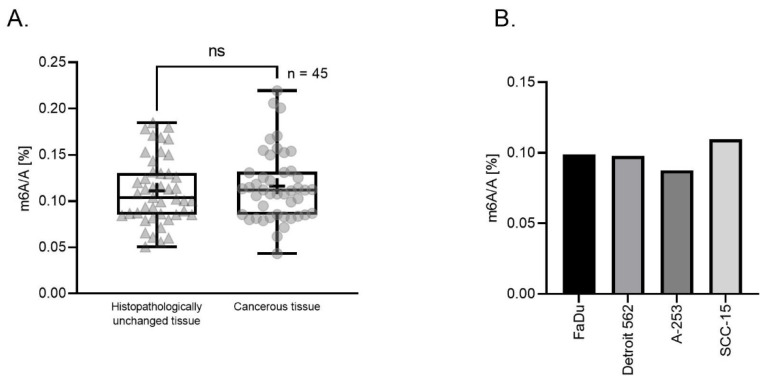
The percentage of m^6^A RNA modification in relation to adenosine (A) in total RNA derived from 45 head and neck squamous cancerous and paired-matched histopathologically unchanged tissues (**A**) and 4 cell lines (FaDu, Detroit 562, A-253, SCC-15) (**B**). ns (non-significant), + (mean value).

We determined the percentage ratio of m^6^A to A in total RNA samples from 45 HNSCC tissues, from 45 adjacent histopathologically unchanged tissues, and in 4 cell lines (FaDu, Detroit 562, A-253, SCC-15 with different tumor locations: hypopharynx, pharynx, salivary gland, and tongue accordingly) using the LC-ESI-MS/MS method. We have observed no significant differences in m^6^A level comparing both types of tissues in the examined HNSCC patients’ group (median value is 0.1037 for histopathologically unchanged tissues and 0.1119 for cancerous tissues, respectively) (Figure 1A). We have also characterized the m^6^A level in the HNSCC cell lines with mean values equaling: FaDu 0.099, Detroit 562 0.098, A-253 0.087, and SCC-15 0.110 (Figure 1B), with an overall mean value μ = 0.098, SD = 0.008. 

Furthermore, we have evaluated the association between m^6^A level and patients’ clinical features, and we have observed no significant differences in m^6^A level between cancerous and paired-matched histopathologically unchanged tissues from all examined HNSCC patients according to patient age at time of surgery, gender, primary tumor size, histological grade, advancement of lymph node metastases, and anatomical site (Table S2). 

### 3.2. mRNA Levels of METTL3 and FTO Genes in HNSCC Patient Samples and Cell Lines

**Figure 2:**
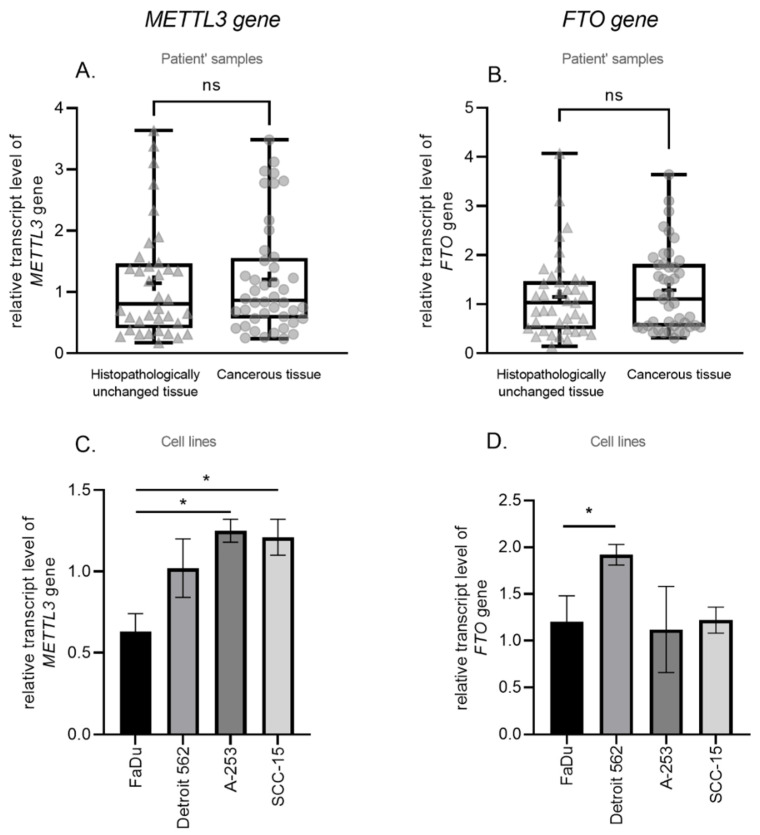
The transcript level of (**A**) *METTL3* and (**B**) *FTO* genes in cancerous and histopathologically unchanged tissues from HNSCC patients and (**C**,**D**) cell lines. ns (non-significant), + (mean value), * (*p* ≤ 0.05).

**Figure 3:**
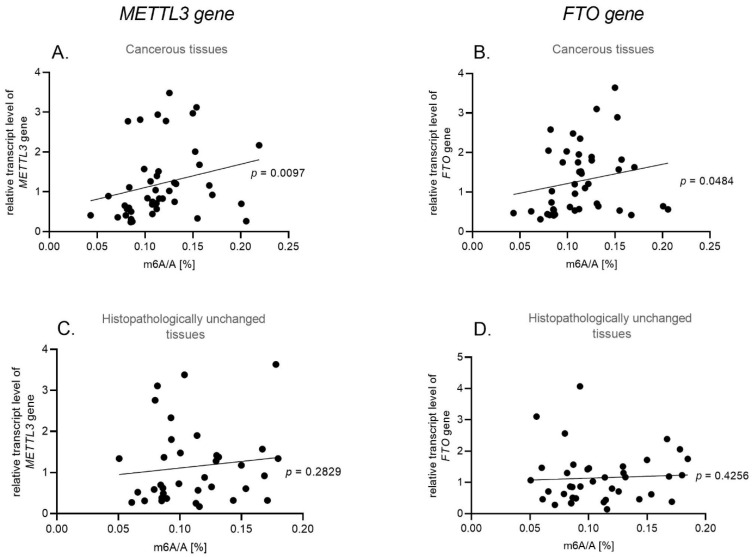
Spearman correlation of m^6^A/A [%] and *METTL3* and *FTO* mRNA level in cancerous tissues (**A**,**B**) and histopathologically unchanged tissues (**C**,**D**).

We have used qPCR to determine the *METTL3* (methyltransferase) and *FTO* (demethylase) transcript levels in cancerous and paired-matched histopathologically unchanged tissues from 45 patients with HNSCC and in 4 cell lines (FaDu, Detroit 562, A-253, SCC-15). We have found no significant differences in *METTL3* (*p* = 0.6494) and *FTO* (*p* = 0.3611) mRNA levels between those groups (Figure 2A,B) and no correlation with clinical features (Tables S3 and S4). Moreover, we have performed the Kaplan–Meier survival analysis among HNSCC patients according to the increased/decreased mRNA level of *METTL3* and *FTO* genes in cancerous tissues compared to paired-matched histopathologically unchanged tissues, and we have not observed a statistically significant difference in the probability of survival for any of the analyzed genes (Figure S2).The FaDu cell line, with a 0.62 value of *METTL3* relative expression, statistically differs from A-253 with a 1.25 *METTL3* expression value (*p* = 0.02) and SCC-15 with a 1.21 *METTL3* expression value (*p* = 0.036) (Figure 2C). In the case of the *FTO* transcript level, the Detroit 562 cell line has a statistically significant higher relative expression value (1.92) than FaDu (1.17) (*p* = 0.033) (Figure 2D). Additionally, we have observed a statistically significant correlation between m^6^A level and *METTL3* and *FTO* transcript levels in cancerous tissue (*p* = 0.0097, r = 0.3856; *p* = 0.0484, r = 0.3028, respectively) (Figure 3A,B) and no correlation in histopathologically unchanged tissue (Figure 3C,D).

### 3.3. mRNA Levels of m^6^A Readers YTHDF2 and YTHDC2 Genes in HNSCC Patient Samples and Cell Lines

**Figure 4:**
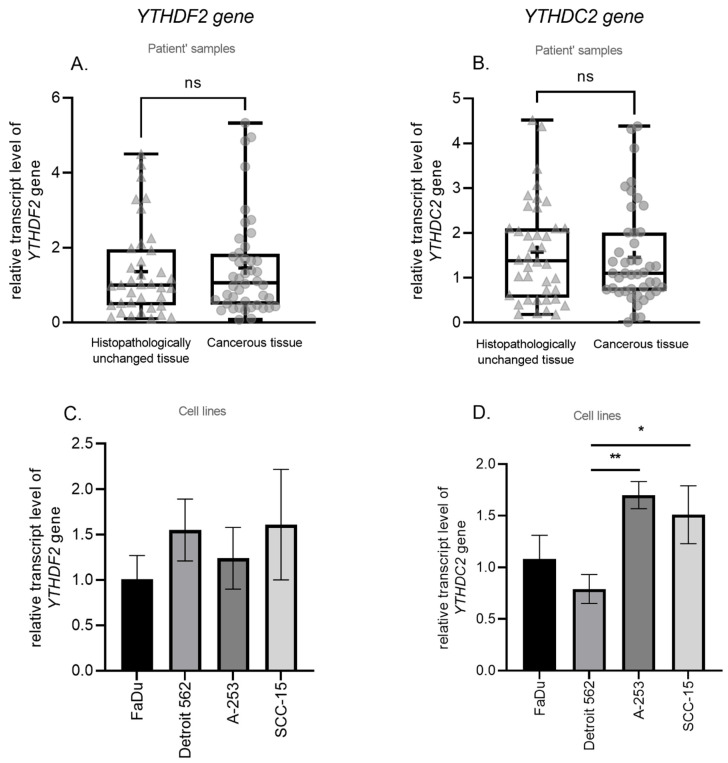
The transcript level of (**A**) *YTHDF2* and (**B**) *YTHDC2* genes in cancerous and histopathologically unchanged tissues from patients with HNSCC and cell lines (**C**,**D**). ns (non-significant), + (mean value), * (*p* ≤ 0.05), ** (*p* < 0.01).

**Figure 5:**
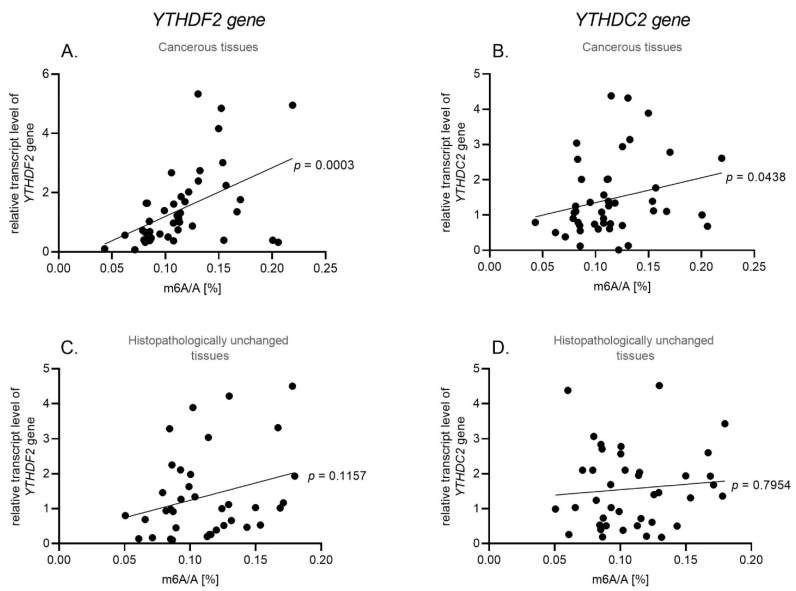
Spearman correlation of m^6^A/A [%] and *YTHDF2* and *YTHDC2* mRNA level in cancerous tissues (**A**,**B**) and histopathologically unchanged tissues (**C**,**D**).

We have used qPCR to compare *YTHDF2* and *YTHDC2* (m^6^A readers) transcript levels in cancerous and paired-matched histopathologically unchanged tissues from 45 patients with HNSCC and in 4 cell lines (FaDu, Detroit 562, A-253, SCC-15). We found no significant differences in mRNA levels between cancerous and histopathologically unchanged tissues (Figure 4A,B) and no correlation with clinical features (Tables S5 and S6). Moreover, we have performed the Kaplan–Meier survival analysis among the HNSCC patients according to the increased/decreased mRNA level of *YTHDF2* and *YTHDC2* genes in cancerous tissues compared to paired-matched histopathologically unchanged tissues, and we have not observed any statistically significant difference in the probability of survival for any of the analyzed genes (Figure S3). The *YTHDF2* transcript level does not significantly differ in all studied cell lines in which FaDu corresponds to the lowest value of the *YTHDF2* relative expression (1.01) and SCC-15 to the highest (1.61) (Figure 4C). In the case of the *YTHDC2* transcript level, the Detroit 562 cell line has a statistically significant lower relative expression value (0.79) than A-253 (1.70) (*p* = 0.001) and SCC-15 (1.51) (*p* = 0.029) (Figure 4D). Moreover, we have observed a statistically significant correlation between m^6^A level and *YTHDF2* and *YTHDC2* transcript levels in cancerous tissue (*p* = 0.0003, r = 0.5170; *p* = 0.0438, r = 3053, respectively) (Figure 5A,B) and no correlation in histopathologically unchanged tissue (Figure 5C,D). 

## 4. Discussion

Accumulating data indicates the significant role of m^6^A RNA modification in the post-transcriptional regulation of gene expression that determines the proper function of crucial physiological processes. Therefore, disturbances in m^6^A RNA methylation may lead to the development of pathological changes, including cancers [11,12,13,14,15,16,17,18,19,20,21,22]. HNSCC initiation, progression, and growth are complex molecular processes encompassing genetic and epigenetic alterations. Unfortunately, the potential association between m^6^A RNA methylation and HNSCC development is poorly described. Thus, the primary goal of this study was to quantify the m^6^A modification in total RNA and the expression of m^6^A RNA methylation-related genes in HNSCC patients and cell lines. Analyzing the study cohort of 45 HNSCC patients with differential clinical features, we have not observed differences in the amount of m^6^A RNA modification nor a correlation with clinical features. However, to the best of our knowledge, we have determined for the first time the m^6^A modification level in four HNSCC cell lines. To date, a similar m^6^A quantification method was used in ovarian cancer studies that also showed no differences between ovarian cancer and normal tissues but indicated a correlation between its elevated content with patients’ overall survival [21]. In the case of pancreatic cancer, the higher level of m^6^A modification measured using HPLC/MS was also correlated with patients’ overall survival and with more lymphatic metastases [22]. Among head and neck cancers, the higher level of m^6^A RNA modification in tumor tissues was found in 34 laryngeal squamous cell carcinoma (LSCC) and paired-matched adjacent normal tissues [14]. However, the measurements were done with a colorimetric method that is less sensitive than LC-ESI-MS/MS. Other studies analyzed the occurrence of m^6^A in specific mRNAs in conjunction with functional consequences on a cellular level [23,24]. Hence, the lack of differences in global m^6^A level between cancerous and histopathologically unchanged tissues in our study cohort does not exclude the role of m^6^A in HNSCC, but rather suggests that m^6^A disturbances in specific mRNA sites may possibly affect HNSCC development. Moreover, we have measured the global m^6^A level that includes a location on almost every type of RNA. As a result, the final picture is more complex, as disturbances of m^6^A quantity may differ depending on their specific location, as opposed to being solely mRNA related. Notably, with regard to the crucial role of m^6^A modification in shaping and controlling the tumor microenvironment (TME), its distribution in the tumor may vary in terms of hypoxia, metabolic dysregulation, immune escape, and chronic inflammation and, as a result, also be strongly dependent on cellular conditions [13]. 

The m^6^A RNA methylation process is regulated by the interaction between components of the methylation complex (METTL3, METTL14, WTAP, RBM15, and VIRMA protein), demethylases (FTO, ALKBH5) that remove methyl groups, and enzymes that bind to m^6^A modification (YTHDF1, YTHDF2, YTHDF3, YTHDC1, and YTHDC2). Thus, we have also measured the mRNA level of selected RNA methylation-related genes (*METTL3*, *FTO*, *YTHDF2,* and *YTHDC2*), which were noticed to significantly affect the tumorigenesis process, and we have determined their correlation with m^6^A status and HNSCC patients’ clinical features and overall survival. Zhao and Cui indicated the differentially expressed m^6^A RNA methylation regulators in HNSCC, based on the TCGA database, and validated these results in 236 HNSCC patients. They have found increased transcript levels of *METTL3*, *YTHDF1*, *VIRMA*, *ALKBH5*, *YTHDF2*, *METTL14*, *WTAP*, and *RBM15* genes and a decreased level of the *YTHDC2* gene [15]. 

Previous studies have shown that the catalytic core enzyme METTL3 plays critical roles in a variety types of cancer as an oncogene and a tumor suppressor. For instance, the overexpression of METTL3 and METTL14 correlates with advanced T stage and poor patient prognosis in HNSCC tissue samples [17]. The *METTL3* gene was also found to be upregulated in oral squamous cell carcinoma (OSCC) patients from TCGA database analysis and independent cancerous clinical samples [18]. Following this, in vitro and in vivo studies have shown that *METTL3* knockdown reduces OSCC cell proliferation, self-renewal, migration, and invasion [18]. Subsequently, m^6^A RNA methylation erasers, FTO and ALKBH5, were identified to be dysregulated in HNSCC and other cancers [25,26,27,28]. In our study cohort, we have not observed the higher transcript level of the *METTL3* and *FTO* genes in both examined tissue types. However, we have found a positive correlation between their expression and the total m^6^A abundance in cancerous tissues. Additionally, we have not noticed that lower m^6^A modification content is correlated with a decreased mRNA level of the *FTO* gene, but conversely, the more m^6^A modifications, the higher FTO expression. These findings correspond with other researchers’ group results [28,29]. This could be explained by the FTO preference to demethylate 2-*O*-dimethyladenosine (m^6^A_m_) and *N*^1^-methyladenosine (m^1^A) rather than m^6^A. The last group of analyzed genes (*YTHDF2*, *YTHDC2*) belongs to the m^6^A RNA methylation readers family. YTHDF2 binding to m^6^A promotes RNA decay, while YTHDC2 is involved in mRNA degradation and translation initiation. These regulators were demonstrated to play a crucial role in a variety types of cancers [21,30,31,32]. The role of the *YTHDF2* gene in HNSCC has not been explored so far, whereas *YTHDC2* was found to be a tumor suppressor with low expression in HNSCC samples, while its upregulation was associated with longer-term survival and the recurrence-free survival of HNSCC patients [33]. In our study, we have observed a statistically significant positive correlation between mRNA levels of *YTHDF2* and *YTHDC2* genes and the relative m^6^A modification amount in cancerous tissues. Even though we have not observed the overall differences in the mRNA expression level of selected genes between cancerous and paired-matched histopathologically unchanged tissues in our patients’ cohort, the correlation of selected genes’ expression with the total m^6^A abundance may suggest the relevance of m^6^A RNA methylation machinery in HNSCC. Moreover, we have also indicated the differences in METTL3, FTO, and YTHDC2 mRNA levels in HNSCC cell lines. The METTL3 transcript amount significantly differs between the FaDu cell line (hypopharynx localization) and the A-253 (salivary glands) as well as SCC-15 (tongue). Moreover, in OSCC tissues, the *METTL3* gene was found to be upregulated [18] similar to the OSCC in vitro model SCC-15 in our results. Subsequently, the FaDu cell line exhibits a significantly lower FTO mRNA level compared with Detroit-562 (pharynx localization). Finally, Detroit-562 corresponds with the lowest expression of the m^6^A reader protein YTHDC2.

The limitation of our study is a quite small study group and a lack of description of the molecular mechanisms and consequences for tumorigenesis in HNSCC in vitro or in vivo models. Moreover, a more detailed study is needed to quantify the m^6^A abundance not only in total RNA but also in mRNA specifically. However, our future studies aim for the high-throughput identification of m^6^A modification sites (MeRIP-seq) and the same identification the genes of its significantly higher abundance and for the performance of in vitro studies to estimate its functional effect on HNSCC cells. Such an examination is required to better understand the RNA methylation impact on HNSCC tumorigenesis. Furthermore, due to patients’ material limitations, we have not determined the protein level of the selected RNA methylation-related components.

## 5. Conclusions

Our study indicates the positive correlation of total RNA m^6^A modification abundance with selected methyltransferase, demethylase, and binding proteins. Moreover, the lack of global m^6^A differences between cancerous and histopathologically unchanged tissues suggests that the final picture could be blurred by our approach, in which we have measured the m^6^A level in total RNA, whereas alterations of m^6^A at individual mRNA possibly influence HNSCC tumorigenesis. Additionally, the differences in mRNA levels of METTL3, FTO, and YTHDC2 in the studied cell lines indicate that its expression may vary depending on the tumor localization. Future perspectives encompass the identification of m^6^A sites by high-throughput analysis (MeRIP-seq) and the estimation of the functional effects of HNSCC cells.

## Figures and Tables

**Table 1:** Characteristics of the study cohort.

Characteristic	Total Number (n/%)
Total study cohort	90
Histopathologically unchanged tissues	45
Cancerous tissues	45
Age at the time of surgery (years)	
Mean	60
Median	61.5
Range	26–91
Gender [n/(%)]	
Male	30 (66.77)
Female	15 (33.33)
Tumor stage (TNM classification) [n/(%)]	
T1	3 (6.67)
T2	14 (31.11)
T3	10 (22.22)
T4	16 (35.56)
N0	16 (35.55)
N1	8 (17.78)
N2	13 (28.89)
N3	3 (6.67)
Histologic grade [n/(%)]	
G1	6 (13.33)
G2	33 (73.33)
G3	6 (13.33)
Anatomical site [n/(%)]	
Larynx	17 (37.78)
Oral cavity	25 (55.55)
Oropharynx	3 (6.67)

## Data Availability

Data supporting reported results are available from the first author to all interested researchers.

## Supplementary Materials

The following are available online at https://www.mdpi.com/article/10.3390/biom11060908/s1, Figure S1: Standard curve of methylation level m^6^A/A [%], Figure S2: The Kaplan-Meier survival analysis of HNSCC patients according to increased/decreased mRNA level of METTL3 (A) and FTO (B) genes in cancerous tissues compared to histopathologically unchanged tissues, Figure S3: The Kaplan-Meier survival analysis of HNSCC patients according to increased/decreased mRNA level of YTHDF2 (A) and YTHDC2 (B) genes in cancerous tissues compared to histopathologically unchanged tissues; Table S1: Primers sequences used in qPCR analysis, Table S2: m^6^A/A ratio in cancerous and histopathologically unchanged tissue samples from patients with HNSCC, Table S3: *METTL3* transcript levels in cancerous and histopathologically unchanged tissue samples from patients with HNSCC, Table S4: *FTO* transcript levels in cancerous and histopathologically unchanged tissue samples from patients with HNSCC, Table S5: *YTHDF2* transcript levels in cancerous and histopathologically unchanged tissue samples from patients with HNSCC, Table S6: *YTHDC2* transcript levels in cancerous and histopathologically unchanged tissue samples from patients with HNSCC.

## Abbreviations

ALKBH5	AlkB homolog 5
FTO	Fat mass and obesity associated protein
HNSCC	Head and neck squamous cell carcinoma
LC-ESI-MS/MS	Liquid chromatography electrospray ionization tandem mass spectrometric
LSCC	Laryngeal squamous cell carcinoma
m^1^A	*N*^1^-methyladenosine
m^6^A	*N*^6^-methyladenosine
m^6^A_m_	2-*O*-dimethyladenosine
METTL3, 14	Methyltransferase like 3, 14
OSCC	Oral squamous cell carcinoma
qPCR	Quantitavie polymerase chain reaction
RBM15	RNA-binding motif protein 15
TCGA	The Cancer Genome Atlas
TME	Tumor microenvironment
WTAP	WT1 associated protein
VIRMA	Vir-like m^6^A methyltransferase associated
YTHDC1, 2	YTH domain containing 1, 2
YTHDF1, 2, 3	*N*^6^-metyladenosine RNA binding protein 1, 2, 3
