# Supplementary material for: The m6A RNA Modification Quantity and mRNA Expression Level of RNA Methylation-Related Genes in Head and Neck Squamous Cell Carcinoma Cell Lines and Patients
Source: Biomolecules. 2021 Jun 18;11(6):908. doi: 10.3390/biom11060908 (PMC8235215; doi:10.3390/biom11060908)
Supplement: Supplementary file 1 [file biomolecules-11-00908-s001.zip › biomolecules-1252615-supplementary.pdf]

Supplementary Materials

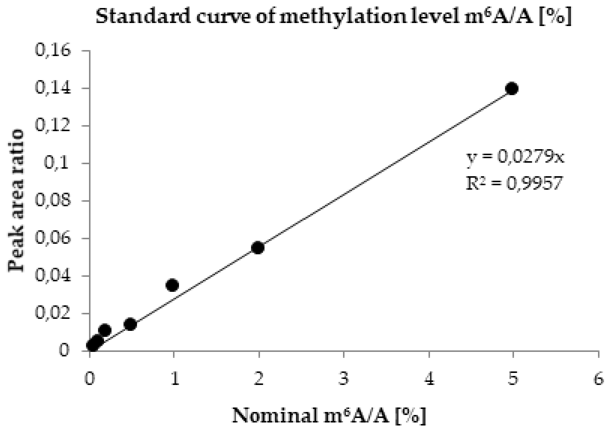

Figure S1. Standard curve of methylation level m6A/A [%].

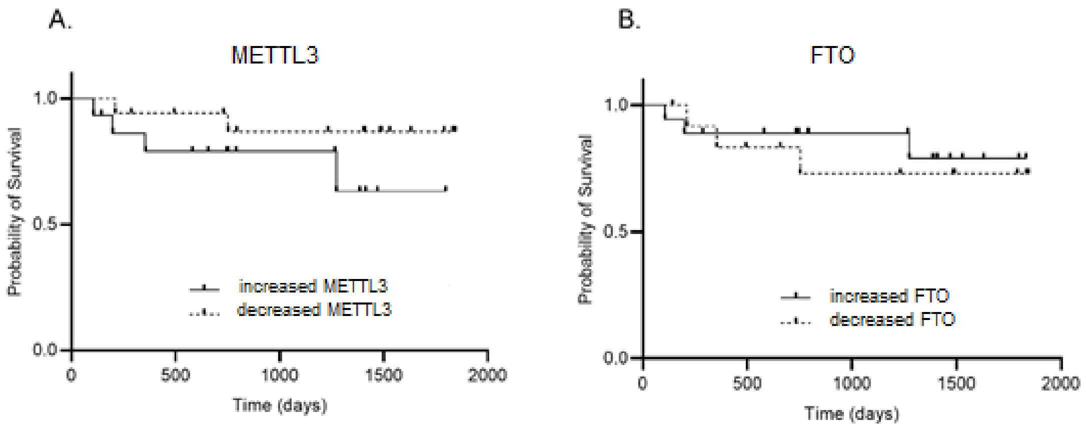

Figure S2. The Kaplan-Meier survival analysis of HNSCC patients according to increased/ decreased mRNA level of METTL3 (**A**) and FTO (**B**) genes in cancerous tissues compared to histopathologically unchanged tissues.

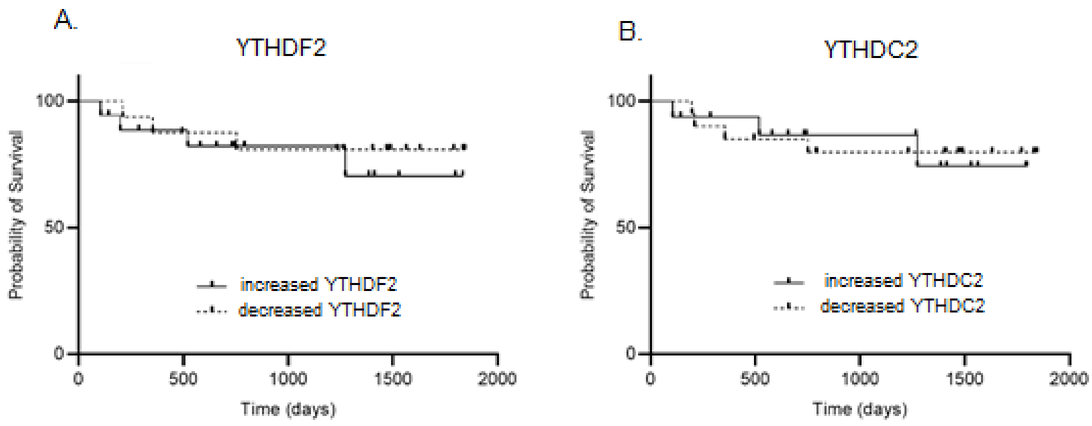

Figure S3. The Kaplan-Meier survival analysis of HNSCC patients according to increased/ decreased mRNA level of YTHDF2 (**A**) and YTHDC2 (**B**) genes in cancerous tissues compared to histopathologically unchanged tissues.

Table S1. Primers sequnce used in qPCR analysis.

Gene	Sequence (5′-3′)
GAPDH	F: GTCTCCTCTGACTTCAACAGCG<br>R: ACCACCCTGTTGCTGTAGCCAA
METTL3	F: TTTTCCGGTTAGCCTTCGGG<br>R: GATAGAGCTCCACGTGTCCG
FTO	F: ACTTGGCTCCCTTATCTGACC<br>R: TGTGCAGTGTGAGAAAGGCTT
YTHDF2	F: AACTGCGACACATTCGCCTA<br>R: TCTCTGGTTCCTCCTTCCCTAA
YTHDC2	F: TTGGATGAGGGCCTTTGGTTT<br>R: CCATTGGTCCTTGTTGCTCAC

F- forward primer

R- reverse primer

Table S2. m^6A/A ratio in cancerous and histopathologically unchanged tissue samples from patients with HNSCC

Characteristic	Cancerous tissue	Histopathologically unchanged tissue	p-value
	Median (range) [a] or Mean (± SD) [b]	Median (range) [a] or Mean (± SD) [b]	
**Age at the time of surgery (years)**			
?60	0.11 ± 0.04 [b]	0.11 ± 0.04 [b]	0.74
>60	0.12 ± 0.04 [b]	0.11 ± 0.04 [b]	0.56
**Gender**			
Male	0.11 ± 0.03 [b]	0.11 ± 0.03 [b]	0.86
Female	0.13 ± 0.04 [b]	0.11 ± 0.04 [b]	0.23
**Tumor stage (TNM classification)**			
T1	0.12 ± 0.05 [b]	0.14 ± 0.05 [b]	0.64
T2	0.13 ± 0.05 [b]	0.11 ± 0.04 [b]	0.20
T3	0.10 ± 0.03 [b]	0.10 ± 0.02 [b]	0.82
T4	0.11 [0.08-0.21] [a]	0.11 [0.06-0.18] [a]	0.90
N0	0.11 [0.08-0.22] [a]	0.11 [0.06-0.17] [a]	0.71
N1	0.12 ± 0.04 [b]	0.12 ± 0.03 [b]	0.88
N2	0.12 ± 0.05 [b]	0.13 ± 0.04 [b]	0.76
N3	0.078 ± 0.03 [b]	0.084 ± 0.03 [b]	0.83
**Histologic grade**			
G1	0.14 [0.08-0.20] [a]	0.11 [0.09-0.18] [a]	0.48
G2	0.11 [0.04-0.22] [a]	0.11 [0.05-0.18] [a]	0.87
G3	0.11 [0.08-0.17] [a]	0.09 [0.06-0.18] [a]	0.39
**Anatomical site**			
Larynx	0.11 [0.08-0.17] [a]	0.10 [0.07-0.18] [a]	0.71
Oral cavity	0.11 [0.04-0.22] [a]	0.11 [0.05-0.18] [a]	0.95
Oropharynx	0.13 ± 0.02 [b]	0.09 ± 0.03 [b]	0.12

Depending on the data distribution we performed the U Mann-Whitney test[a] or unpaired t-test with Welch's correction [b].

Table S3. *METTL3* transcript levels in cancerous and histopathologically unchanged tissue samples from patients with HNSCC.

Characteristic	Cancerous tissue	Histopathologically unchanged tissue	p-value
	Median (range) [a] or Mean (± SD) [b]	Median (range) [a] or Mean (± SD) [b]	
**Age at the time of surgery (years)**			
?60	0.84 [0.24-2.90] [a]	0.92 [0.17-3.63] [a]	0.89
>60	0.89 [0.26-3.48] [a]	0.70 [0.31-3.38] [a]	0.40
**Gender**			
Male	0.83 [0.26-2.97] [a]	0.70 [1.7-3.38] [a]	0.73
Female	1.65 ± 1.17 [b]	1.38 ± 1.14 [b]	0.56
**Tumor stage (TNM classification)**			
T1	1.67 ± 1.62 [b]	2.44 ± 1.38 [b]	0.57
T2	1.36 [0.41-3.12] [a]	1.31 [0.17-2.33] [a]	0.68
T3	0.65 [0.25-1.51] [a]	0.57 [0.31-3.38] [a]	0.71
T4	0.83 [0.24-2.97] [a]	0.67 [0.25-3.11] [a]	>0.99
N0	0.76 [0.33-3.48] [a]	0.92 [0.31-2.76] [a]	0.93
N1	1.02 [0.24-2.94] [a]	0.84 [0.27-3.36] [a]	0.75
N2	1.43 ± 0.96 [b]	1.25 ± 0.97 [b]	0.66
N3	0.54 ± 0.12 [b]	0.67 ± 0.60 [b]	0.76
**Histologic grade**			
G1	0.76 [0.57-3.12] [a]	0.73 [0.17-1.80] [a]	0.93
G2	0.90 [0.24-3.48] [a]	1.28 [0.25-3.38] [a]	0.90
G3	0.71 ± 0.39 [b]	0.49 ± 0.10 [b]	0.34
**Anatomical site**			
Larynx	0.84 [0.25-2.97] [a]	0.63 [0.25-3.38] [a]	0.98
Oral cavity	0.95 [0.24-3.48] [a]	0.90 [0.27-3.63] [a]	0.76
Oropharynx	1.48 ± 1.42 [b]	0.98 ± 1.15 [b]	0.80

The *METTL3* transcript levels were measured in triplicates and standardized by *GAPDH* reference gene, relative gene expression were calculate using Pfaffl method. Depending on the data distribution we performed the U Mann-Whitney test[a] or unpaired t-test with Welch's correction[b].

Table S4. *FTO* transcript levels in cancerous and histopathologically unchanged tissue sample from patient with HNSCC.

Characteristic	Cancerous tissue	Histopathologically unchanged tissue	p-value
	Median (range) [a] or Mean (± SD) [b]	Median (range) [a] or Mean (± SD) [b]	
Age at the time of surgery (years)			
?60	1.06 [0.31-3.64] [a]	1.65 [0.14-4.07] [a]	0.66
>60	1.20 [0.41-3.10] [a]	0.87 [0.33-3.10] [a]	0.48
Gender			
Male	1.15 [0.31-3.64] [a]	0.95 [0.14-3.10] [a]	0.13
Female	0.96 [0.41-2.58] [a]	1.17 [0.46-4.07] [a]	0.55
Tumor stage (TNM classification)			
T1	0.84 ± 0.83 [b]	1.94 ± 0.69 [b]	0.16
T2	1.47 ± 0.76 [b]	1.61 ± 1.10 [b]	0.72
T3	0.53 [0.42-1.52] [a]	0.51 [0.33-1.45] [a]	0.71
T4	1.48 ± 0.97 [b]	0.99 ± 0.60 [b]	0.10
N0	0.59 [0.42-2.35] [a]	1.03 [0.34-2.06] [a]	0.29
N1	1.34 [0.41-3.64] [a]	1.16 [0.33-4.07] [a]	0.92
N2	1.41 ± 0.89 [b]	1.12 ± 0.59 [b]	0.46
N3	0.49 ± 0.07 [b]	0.57 ± 0.47 [b]	0.80
Histologic grade			
G1	1.07 ± 0.52 [b]	1.12 ± 0.62 [b]	0.88
G2	1.47 [0.31-3.64] [a]	1.11 [0.28-4.07] [a]	0.15
G3	0.54 [0.43-0.71] [a]	0.57 [0.33-3.10] [a]	>0.99
Anatomical site			
Larynx	1.47 [0.42-3.64] [a]	0.78 [0.28-2.38] [a]	0.17
Oral cavity	1.10 [0.31-2.89] [a]	1.16 [0.34-4.07] [a]	0.85
Oropharynx	0.95 ± 0.54 [b]	1.37 ± 1.54 [b]	0.69

The *FTO* transcript levels were measured in triplicates and standardized by *GAPDH* reference gene, relative gene expression were calculate using Pfaffl method. Depending on the data distribution we performed the U Mann-Whitney test[a] or unpaired t-test with Welch's correction[b].

Table S5. *YTHDF2* transcript levels in cancerous and histopathologically unchanged tissue sample from patient with HNSCC.

Characteristic	Cancerous tissue	Histopathologically unchanged tissue	p-value
	Median (range) [a] or Mean (± SD) [b]	Median (range) [a] or Mean (± SD) [b]	
Age at the time of surgery (years)			
?60	0.87 [0.07-4.95] [a]	0.92 [0.14-4.50] [a]	0.79
>60	1.39 [0.32-5.33] [a]	1.10 [0.10-4.22] [a]	0.89
Gender			
Male	1.00 [0.07-5.33] [a]	0.99 [0.10-4.22] [a]	0.84
Female	1.35 [0.38-4.95] [a]	1.03 [0.14-4.50] [a]	0.76
Tumor stage (TNM classification)			
T1	0.71 ± 0.90 [b]	2.76 ± 2.46 [b]	0.38
T2	1.88 ± 1.24 [b]	1.60 ± 1.24 [b]	0.64
T3	0.60 [0.32-2.74] [a]	0.52 [0.10-1.98] [a]	0.80
T4	1.01 [0.32-5.33] [a]	1.00 [0.14-4.22] [a]	0.99
N0	1.10 [0.07-5.33] [a]	1.27 [0.17-4.22] [a]	0.56
N1	1.00 [0.32-2.24] [a]	1.04 [0.13-4.50] [a]	0.95
N2	1.22 [0.38-4.95] [a]	0.98 [0.20-3.32] [a]	0.82
N3	0.67 ± 0.54 [b]	0.39 ± 0.37 [b]	0.50
Histologic grade			
G1	1.69 ± 0.89 [b]	1.29 ± 0.74 [b]	0.43
G2	0.80 [0.07-5.33] [a]	0.94 [0.13-4.22] [a]	0.74
G3	1.58 ± 1.15 [b]	0.85 ± 0.67 [b]	0.34
Anatomical site			
Larynx	1.31 [0.32-5.33] [a]	0.98 [0.10-4.22] [a]	0.75
Oral cavity	0.92 [0.07-4.95] [a]	1.01 [0.13-4.50] [a]	0.75
Oropharynx	2.19 ± 0.93 [b]	0.76 ± 0.71 [b]	0.15

The *YTHDF2* transcript levels were measured in triplicates and standarized by *GAPDH* reference gene, relative gene expression were calculate using Pfaffl method. Depending on the data distribution we performed the U Mann-Whitney test[a] or unpaired t-test with Welch's correction[b].

Table S6. *YTHDC2* transcript levels in cancerous and histopathologically unchanged tissue sample from patient with HNSCC.

Characteristic	Cancerous tissue<br>Median (range) [a] or<br>Mean (± SD) [b]	Histopathologically unchanged tissue<br>Median (range) [a] or<br>Mean (± SD) [b]	p-value
Age at the time of surgery (years)			
?60	0.93 [0.01-3.89] [a]	1.36 [0.18-4.38] [a]	0.29
>60	1.18 [0.12-4.38] [a]	1.40 [0.19-4.52] [a]	0.82
Gender			
Male	0.98 [0.13-4.38] [a]	1.46 [0.19-4.52] [a]	0.25
Female	1.37 [0.01-3.04] [a]	1.03 [0.18-4.38] [a]	0.62
Tumor stage (TNM classification)			
T1	1.47 ± 1.32 [b]	2.12 ± 0.87 [b]	0.52
T2	1.42 ± 0.88 [b]	1.46 ± 1.23 [b]	0.92
T3	1.11 [0.50-3.14] [a]	2.04 [0.19-2.84] [a]	0.51
T4	0.76 [0.12-4.38] [a]	1.32 [0.26-4.52] [a]	0.75
N0	1.26 [0.38-4.32] [a]	1.93 [0.38-4.52] [a]	0.61
N1	0.70 [0.50-1.77] [a]	1.04 [0.26-2.10] [a]	0.75
N2	1.72 ± 1.43 [b]	1.66 ± 1.21 [b]	0.92
N3	1.03 ± 0.32 [b]	0.63 ± 0.41 [b]	0.26
Histologic grade			
G1	1.58 ± 0.55 [b]	1.46 ± 0.84 [b]	0.80
G2	0.98 [0.01-4.98] [a]	1.57 [0.18-4.52] [a]	0.47
G3	1.54 ± 1.31 [b]	1.71 ± 1.37 [b]	0.88
Anatomical site			
Larynx	1.26 [0.55-4.38] [a]	2.10 [0.19-4.52] [a]	0.84
Oral cavity	0.95 [0.01-2.94] [a]	1.31 [0.18-4.38] [a]	0.70
Oropharynx	0.97 ± 0.73 [b]	0.87 ± 0.22 [b]	0.85

The *YTHDC2* transcript levels were measured in triplicates and standarized by *GAPDH* reference gene, relative gene expression were calculate using Pfaffl method. Depending on the data distribution we performed the U Mann-Whitney test[a] or unpaired t-test with Welch's correction[b].
